# Rest tremor in Parkinson’s disease: the theta and beta sides of the coin

**DOI:** 10.1093/braincomms/fcaa128

**Published:** 2020-08-18

**Authors:** Guglielmo Foffani, Mariana H G Monje, Jose A Obeso

**Affiliations:** HM-CINAC, Hospital Universitario HM Puerta del Sur, Universidad CEU-San Pablo, Móstoles, Madrid, Spain; Hospital Nacional de Parapléjicos, Toledo, Spain; CIBERNED, Instituto de Salud Carlos III, Madrid, Spain; HM-CINAC, Hospital Universitario HM Puerta del Sur, Universidad CEU-San Pablo, Móstoles, Madrid, Spain; HM-CINAC, Hospital Universitario HM Puerta del Sur, Universidad CEU-San Pablo, Móstoles, Madrid, Spain; CIBERNED, Instituto de Salud Carlos III, Madrid, Spain

## Abstract

This scientific commentary refers to ‘Independently together: subthalamic theta and beta opposite roles in predicting Parkinson’s tremor’, by Asch *et al.* (https://doi.org/10.1093/braincomms/fcaa074).

**This scientific commentary refers to ‘Independently together: subthalamic theta and beta opposite roles in predicting Parkinson’s tremor’, by Asch *et al.* (https://doi.org/10.1093/braincomms/fcaa074).**

Rest tremor is a paradigmatic manifestation of the ‘shaking palsy’, but more than 200 years after the original essay by James Parkinson, the origin of parkinsonian tremor remains largely enigmatic. A better understanding of parkinsonian tremor is mandatory, particularly because (i) tremor conveys the highest specificity of Parkinson’s disease at onset and (ii) tremor predominant Parkinson’s disease patients typically display a more benign progression than the rigid-akinetic predominant patients.

In this scenario, different clinical features appear to be associated with specific pathophysiological mechanisms. Tremor and rigidity-bradykinesia are nicely separated in the frequency domain: tremor is tautologically associated with neuronal oscillations at tremor frequencies in the theta range (4–7 Hz) (Rodriguez et al., 1998) whereas rigidity-bradykinesia more mysteriously correlates with oscillations at beta frequencies (13–35 Hz) (Little and Brown, 2014). Since slower oscillations require loops with longer propagation delays, this frequency separation suggests that the pathophysiology of tremor versus rigidity-bradykinesia may involve different, possibly interacting, functional networks. In fact, the networks involved in tremor and rigidity-bradykinesia seem to overlap to some extent in the basal ganglia, and certainly in the posterior-dorso-lateral region of the subthalamic nucleus (STN) (Rodriguez-Oroz, 2001). Conveniently, the STN provides a single target for treating all the cardinal motor features of Parkinson’s disease. The overlap is also attractive in terms of pathophysiology because it offers the opportunity to investigate at one anatomical location any possible interactions between the two functional networks involved in tremor and rigidity-bradykinesia.

This opportunity is elegantly captured by Asch *et al.* in a paper published in the present issue of *Brain Communications* (Asch *et al.*, 2020). These authors analysed intraoperative neural recordings from a fairly large dataset of 114 STN in 70 Parkinson’s disease patients undergoing deep brain stimulation (DBS) surgery. They assessed neural activity at theta and beta frequencies and investigated the relationship between these two frequency bands and pre-operative motor signs, as quantified by the Movement Disorder Society-sponsored revision of the Unified Parkinson’s Disease Rating Scale part III (MDS-UPDRS-III). The first important result of their study is that STN theta activity specifically correlates with pre-operative rest tremor but not with action tremor. By ‘action tremor’, the authors refer to both postural tremor, which is common in Parkinson’s disease, and kinetic tremor, which is infrequent. Their results support the notion of functional separation between rest and ‘action’ tremor presentations, which is in line with the classical observation that postural tremor typically—but not always—occurs at higher frequencies than rest tremor (Lance *et al.*, 1963). The authors thus focus on rest tremor, which convincingly appears as an independent motor sign in the clinical parkinsonian spectrum.

The second result to highlight is that pre-operative rest tremor not only correlates positively with STN theta oscillations, which is consistent with previous reports but also shows a tendency toward a negative correlation with STN beta oscillations. A simple metric combining theta and beta activity (i.e. their difference divided by their sum) correlates with tremor better than theta activity alone. This could be related to the observation that rest tremor desynchronizes beta activity (Shreve et *al.*, 2017) and the classic clinical observation that a highly tremoric body segment is not particularly rigid.

But, the situation is actually more complex and more interesting. Theta and beta activities display an overall weak temporal co-activation, but this co-activation correlates negatively with pre-operative resting tremor. In other words, patients whose theta and beta activities fluctuate together in time are associated with less pre-operative rest tremor. This suggests that at least two functionally distinct theta activities may occur in the parkinsonian STN: (i) a more obvious theta activity linked with resting tremor and negatively correlated with beta activity and (ii) a more subtle theta activity displaying amplitude–amplitude coupling with beta activity that is negatively correlated with tremor. The latter may be the same theta activity that was reported to display phase–phase coupling in rigid-akinetic Parkinson’s disease patients (Marceglia *et al.*, 2006) and to increase after levodopa administration, particularly in the presence of levodopa-induced dyskinesias (Alonso-Frech *et al.*, 2006). Accordingly, STN in Parkinson’s disease exhibits a more complex range of oscillatory theta activity than the tremor-related kind.

As a last paramount result, the authors show that a multispectral regression model integrating theta activity, beta activity and their temporal co-activation nicely provides the best predictor of pre-operative rest tremor. Therefore, the possible feasibility and usefulness of the multi-spectrum model as a biomarker for adaptive DBS will crucially depend on the ability of the model to predict the temporal fluctuations of rest tremor in patients (and to do it better than theta activity alone). This should be tested in future studies. Independently of their outcome, the proposed multi-spectrum model already provides an elegant new framework to investigate the pathophysiological footprints of clinical motor subtypes in Parkinson’s disease.

The correlational nature of the cross-sectional study by Asch *et al.* warrants a few final considerations about the intricate relationship between correlation and causality. Under the seemingly reasonable assumption that neural activity causes behaviour, one might be tempted to infer that STN oscillatory activities cause tremor. However, this is not necessarily the case and it may well be that tremor causes STN oscillatory activities, or actually, both may even be true.

Several clinical and pathophysiological features suggest that tremor should be better understood as a *circuitopathy*, which engages somatotopically organized structures beyond the basal ganglia. First, tremor in Parkinson’s disease at the time of diagnosis has a characteristically focal distribution, mainly expressed distally in one limb (i.e. the hand and fingers), and there is no good correlation between the severity of tremor and striatal dopaminergic deficit (Pirker, 2003). On the other hand, there are several brain knots where tremor-related neuronal activity is neatly recorded and blocked by high-frequency electrical stimulation or lesioning. The main ones are the STN and the ventral intermediate nucleus of the thalamus, and to some extent, the globus pallidus pars interna. Furthermore, the activation of the cortico-spinal tract through motor cortex stimulation provokes transient tremor arrest (Ni *et al.*, 2010), which also typically occurs at the beginning of a voluntary movement, and tremor also disappears with lesioning of the pyramidal tract (Browder, 1948).

All of the features summarized above indicate that the abnormal neuronal activity associated with tremor in Parkinson’s disease engages somatotopically organized structures that involve the basal ganglia and the motor cortex. In addition, several neuroimaging studies in recent years have provided large evidence for cerebello-thalamic circuit engagement in Parkinson’s disease tremor (Helmich, 2018). However, to what extent cerebral activity associated with tremor is the cause or consequence is yet to be disentangled.

For the time being, we would favour the idea of a ‘tremor-specific’ circuit mainly mediated by STN-cortical connectivity and secondarily by recruitment of the cerebello-thalamo-cortical circuit. Actually, the focal hand predominance of tremor onset in Parkinson’s disease is quite compatible with a cortically mediated mechanism. Thus, the cortical excitatory connection to the STN, and the reciprocal excitatory–inhibitory effect of the STN and globus pallidus pars externa (GPe) connections could set the necessary excitatory changes in motion producing synchronous and rhythmical neuronal coupling at 4–6 Hz. This could arise as a consequence of excitability changes occurring in the ‘indirect’ circuit associated with dopaminergic striatal depletion, direct dopaminergic loss in the GPe/STN or both (Bevan *et al.*, 2002). Admittedly, there are some difficulties with this proposal. The STN is known to influence thalamo-cortical activity mainly via the globus pallidus pars interna, whose efferent activity is not functionally suited to generate tremor-related activity. Another option would be that the GPe and/or STN would have direct access and capacity to influence thalamic nuclei with high intrinsic and extrinsic capacity to generate rhythmic oscillatory activity like the reticular nucleus (Bazhenov *et al.*, 1999) and the ventral intermediate nucleus of the thalamus (Lenz *et al.*, 1994). However, the functional impact of efferent connections from the GPe/STN to the thalamus has not been demonstrated so far, although specific studies are missing. Finally, one could speculate about the possibility of a direct reciprocal connection between the motor cortex and STN, giving the general dual organization of basal ganglia connectivity. The latter would be a perfect fit for the circuit of Parkinson’s disease tremor, but an STN projection to the cortex has so far not been established.

Clearly, the origin of Parkinson’s disease tremor remains somewhat elusive. To establish causality is a major, highly desirable outcome of any scientific investigation. It is like the holy grail: we should always pursue it, but always doubt when we think we have found it. The positive news is that studies like the one by Asch *et al.* help to advance understanding and we hope also to refine treatment. As in many other aspects of medicine, and certainly as is the case in the therapeutic management of Parkinson’s disease, there is an element of mystery: we manage to obtain an improvement, i.e., stop our patient shaking, but we cannot explain exactly why.

## References

[fcaa128-B1] Alonso-Frech F Zamarbide I Alegre M Rodríguez-Oroz MC Guridi J Manrique M, et al Slow oscillatory activity and levodopa-induced dyskinesias in Parkinson’s disease. Brain 2006; 129: 1748–57. 16684788 10.1093/brain/awl103

[fcaa128-B2] Asch N Herschman Y Maoz R Aurbach-Asch C Valsky D Abu-Snineh M, et al Independently together: subthalamic theta and beta opposite roles in predicting Parkinson’s tremor. Brain Commun 2020; doi: 10.1093/braincomms/fcaa074. 10.1093/braincomms/fcaa074 PMC7869429 33585815

[fcaa128-B3] Bazhenov M Timofeev I Steriade M Sejnowski TJ. Self-sustained rhythmic activity in the thalamic reticular nucleus mediated by depolarizing GABA(A) receptor potentials. Nat Neurosci 1999; 2: 168–74. 10195202 10.1038/5729

[fcaa128-B4] Bevan MD Magill PJ Terman D Bolam JP Wilson CJ. Move to the rhythm: Oscillations in the subthalamic nucleus-external globus pallidus network. Trends Neurosci 2002; 25: 525–31. 12220881 10.1016/s0166-2236(02)02235-x

[fcaa128-B5] Browder J. Section of the fibers of the anterior limb of the internal capsule in parkinsonism. Am J Surg 1948; 75: 264–8. 18918436 10.1016/0002-9610(48)90299-2

[fcaa128-B6] Helmich RC. The cerebral basis of Parkinsonian tremor: a network perspective. Mov Disord 2018; 33: 219–31. 29119634 10.1002/mds.27224

[fcaa128-B7] Lance JW Schwab RS Peterson EA. Action tremor and the cogwheel phenomenon in Parkinson’s disease. Brain 1963; 86: 95–110. 13928399 10.1093/brain/86.1.95

[fcaa128-B8] Lenz FA Kwan HC Martin RL Tasker RR Dostrovsky JO Lenz YE. Single unit analysis of the human ventral thalamic nuclear group. Brain 1994; 117: 531–43. 8032863 10.1093/brain/117.3.531

[fcaa128-B9] Little S Brown P. The functional role of beta oscillations in Parkinson’s disease. Park Relat Disord 2014; 20: S44–S48. 10.1016/S1353-8020(13)70013-0 24262186

[fcaa128-B6715019] Marceglia S Foffani G Bianchi A M Baselli G Tamma F Egidi M, et al Dopamine-dependent non-linear correlation between subthalamic rhythms in Parkinson's disease. The Journal of Physiology 2006; 571: 579–91. 10.1113/jphysiol.2005.100271 16410285 PMC1805793

[fcaa128-B10] Ni Z Pinto AD Lang AE Chen R. Involvement of the cerebellothalamocortical pathway in Parkinson disease. Ann Neurol 2010; 68: 816–24. 21194152 10.1002/ana.22221

[fcaa128-B11] Pirker W. Correlation of dopamine transporter imaging with parkinsonian motor handicap: how close is it? Mov Disord 2003; 18: S43–S51. 10.1002/mds.10579 14531046

[fcaa128-B83946245] Rodriguez MC Guridi OJ Alvarez L Mewes K Macias R Vitek J, et al The subthalamic nucleus and tremor in Parkinson's disease. Mov Disord 1998; 13 Suppl 3: 111–8. 10.1002/mds.870131320 9827606 9827606

[fcaa128-B13] Rodriguez-Oroz MC. The subthalamic nucleus in Parkinson’s disease: somatotopic organization and physiological characteristics. Brain 2001; 124: 1777–90. 11522580 10.1093/brain/124.9.1777

[fcaa128-B15] Shreve LA Velisar A Malekmohammadi M Koop MM Trager M Quinn EJ, et al Subthalamic oscillations and phase amplitude coupling are greater in the more affected hemisphere in Parkinson’s disease. Clin Neurophysiol 2017; 128: 128–37. 27889627 10.1016/j.clinph.2016.10.095

